# *Halobacterium salinarum*: Life with more than a grain of salt

**DOI:** 10.1099/mic.0.001327

**Published:** 2023-04-17

**Authors:** Jerry Eichler

**Affiliations:** ^1^​ Department of Life Sciences, Ben-Gurion University of the Negev, P.O. Box 653, Beersheva 84105, Israel

**Keywords:** Archaea, Halobacterium salinarum, halophiles

## Abstract

*
Halobacterium salinarum* is a halophilic (salt-loving) archaeon that grows in salt concentrations near or at saturation. Although isolated from salted fish a century ago, it was the 1971 discovery of bacteriorhodopsin, the light-driven proton pump, that raised interest in *Hbt. salinarum* across a range of disciplines, including biophysics, chemistry, molecular evolution and biotechnology. *Hbt. salinarum* have since contributed to numerous discoveries, such as advances in membrane protein structure determination and the first example of a non-eukaryal glycoprotein. Work on *Hbt. salinarum*, one of the species used to define Archaea, has also elucidated molecular workings in the third domain. Finally, *Hbt. salinarum* presents creative solutions to the challenges of life in high salt.

## Taxonomy

Domain: *Archaea*; phylum: *Euryarchaeota *; clade: *Stenosarchaea* group; class: *Halobacteria *; order: *Halobacteriales *; family: *Halobacteriaceae *; genus: *Halobacterium *; species: *Halobacterium salinarum. Hbt. salinarum* currently includes six strains, with the type stain being *Hbt. salinarum* strain 91-R6 [1].

## Properties

*Halobacterium salinarum* grows at NaCl concentrations approaching saturation, with high cytosolic K^+^ levels (~4 M) balancing the external Na^+^ concentration [2]. Although an aerobic chemoorganotroph, *Halobacterium salinarum* can also grow anaerobically by relying on bacteriorhodopsin, the light-driven proton pump found in their purple membranes. The *Hbt. salinarum* proteome is highly acidic (average pI ~5), an adaptation that allows haloarchaeal proteins to fold in their hypersaline surroundings [3]. Moreover, as *Halobacterium salinarum* lives in environments characterized by extreme UV radiation and inconsistent nutritional conditions, it displays color-sensitive phototaxis, chemotaxis and aerotaxis [4].

## Genome

The genome of *Hbt. salinarum* strain NRC-1, published in 2000, was the first genome from a halophilic archaeon to be sequenced [5]. The *Halobacterium* NRC-1 genome comprises a 2 Mbp chromosome and two 350 and 200 kbp-containing closed mini-chromosomes, all three of which contain essential genes. The main chromosome presents a high GC content (67.9 %), whereas that of the mini-chromosomes is lower (59.2 and 57.9 %). The large number of transposable insertion sequence elements in the genome (almost 100) explains the observed genetic plasticity of this haloarchaeon. At the time of its release, the *Hbt. salinarum* strain NRC-1 genome was described as encoding 2360 proteins, 36 % of which were deemed as unrelated to any other known proteins at the time. Finally, like other haloarchaea, *Hbt. salinarum* is a polyploid, containing some 25 copies of the genome in exponential phase, yet only 15 copies in stationary phase. Likewise, mini-chromosome copy numbers are also growth phase-regulated.

## Phylogeny

*Hbt. salinarum* was first isolated from salted cod in 1922 but as the original strain was lost, a neotype isolated from salted cowhide in 1934 was designated as the type strain (strain 91-R6). Similar isolates assigned as *Hbt. salinarium*, *Hbt. cutirubrum* or *Hbt. halobium* were eventually deemed to be so closely related that all were subsequently defined as *Hbt. salinarum* species. To date, the genomes of two laboratory strains (*Hbt. salinarum* species NRC-1 and R1), as well as that of the type strain, have been sequenced. A comparison of *Hbt. salinarum* strains NRC-1 and R1 revealed only 12 differences, corresponding to five single-base frameshifts, four point mutations, and three insertion/deletion events, along with differences related to the large number of insertion sequence elements in *Hbt. salinarum* genomes. The *Hbt. salinarum* strain 91-R6 genome shares >99 % identity with the laboratory strain genomes, including only 38 strain-specific regions [1].

## Key features & discoveries

As one of the microorganisms that served to originally define the Archaea, *Hbt. salinarum* has provided novel insight into numerous aspects of this distinct domain of life. The unique ether-linked isoprenoid-based phospholipids that comprise the archaeal plasma membrane were first described in *Hbt. salinarum*. The surface (S)-layer protein comprising the protein shell surrounding *Hbt. salinarum* cells provided the first example of protein N- and O-glycosylation outside the Eukarya [6]. *Hbt. salinarum* was also the source of the first transformation and gene deletion systems developed for Archaea [7]. *Hbt. salinarum*, moreover, provided the first example of a two-component regulatory system in Archaea. It is now known that the phototactic responses of *Hbt. salinarum* rely on such systems. Sensory rhodopsins I and II, together with the transducers HtrI and HtrII, mediate positive and negative phototactic responses to orange and blue light, respectively. At the same time, halorhodopsin drives Cl^−^ uptake in yellow light [8].

Still, it was the 1971 discovery of bacteriorhodopsin [9] that served to draw the attention of researchers from a broad range of disciplines to *Hbt. salinarum*, resulting in several ground-breaking and even paradigm-shifting discoveries. Upon oxygen starvation in the presence of UV light, patches comprising clusters of bacteriorhodopsin trimers organized as a two-dimensional lattice termed purple membrane appear. In such conditions, some 300 000 copies of bacteriorhodopsin are expressed in the cell, corresponding to 75 % of the total membrane dry mass. This arrangement resulted in bacteriorhodopsin being one of the first membrane proteins for which structural information was obtained [10]. The introduction of bacteriorhodopsin into non-native cells eventually led to the development of optogenetics, an experimental approach in which light controls the activity of neurons or other cell types. Indeed, the biotechnological potential of bacteriorhodopsin in bioelectronic applications has long been recognized.

Studies of *Hbt. salinarum* have also enhanced understanding of life in high levels of salt. *Hbt. salinarum* protein surfaces present an excess of negatively-charged residues (i.e. aspartate and glutamate), relative to positively-charged residues (i.e. lysine and arginine), an adaptation thought to allow such proteins to remain soluble and flexible in hypersaline conditions. Intracellular gas vesicles, thought to increase buoyancy in low oxygen-content hypersaline environments, were first observed in *Hbt. salinarum*. Finally, despite encountering high levels of sunlight, *Hbt. salinarum* is exceedingly resistant to UV radiation- and dessication-induced DNA damage, possibly due to the polyploid nature of this microbe.

## Open questions

Why does *Hbt. salinarum* contain so many copies of the genome?

How does *Hbt. salinarum* reversibly respond to transient changes in environmental salinity?

Why does *Hbt. salinarum* require three different pathways of protein glycosylation?

How can the ability of the *Hbt. salinarum* S-layer glycoprotein and bacteriorhodopsin to self-assemble into 2D lattices be exploited biotechnologically?

Will better understanding of how *Hbt. salinarum* copes with hypersalinity and high UV radiation benefit the search for life on other worlds?

## Biography

### Jerry Eichler

Studies post-translational modifications in Archaea, with particular focus on N-glycosylation, using *Halobacterium salinarum *, *Haloferax volcanii* and other haloarchaea as model systems.
